# How Are They Different? Comparison Between Older Adults Who Limit Their Driving And Those Who Cease Driving

**DOI:** 10.1093/geroni/igaf122.2484

**Published:** 2025-12-31

**Authors:** Yunji Han, Yeji Hwang

**Affiliations:** Seoul National University, Seoul, Republic of Korea; Seoul National University, Seoul, Republic of Korea

## Abstract

While some older adults gradually avoid driving long distances or refrain from driving in adverse weather conditions, others completely stop driving. Lack of information is available on the differences between those who restrict their driving and those who completely stop driving. Therefore, this study aimed to identify the differences between older adults who self-restrict their driving and those who completely stop driving. This study employed a secondary data analysis using the Health and Retirement Study data from 2014 to 2018 on individuals who were 60 years and older and who self-restricted their driving at baseline (N = 629). For data analysis, participants were divided into two groups: (1) Those who continued to self-restrict their driving from 2014 to 2018 (n = 502, 79.8%), and (2) those who self-restricted their driving in 2014 but completely ceased driving in 2018 (n = 127, 20.2%). Chi-square tests and independent t-tests were performed to evaluate the differences between the two groups. The ceased-driving group were more likely to have a higher prevalence of dementia (χ²=5.880, p = 0.015) or depression (χ²=4.012, p = 0.045). Additionally, there was a significant difference in subjective health (t = 2.194, df = 626, p = 0.029), with the ceased-driving group reporting poor subjective health scores. The findings showed that the transition from limited driving to complete driving cessation in older adults is related to one’s cognitive function, psychological health and subjective health. Attentions are needed for older adults who have such conditions to delay complete driving cessation and to support them with substitute measures for transportation.

